# Epilepsy and Cognitive Impairment in Childhood and Adolescence: A Mini-Review

**DOI:** 10.2174/1570159X20666220706102708

**Published:** 2023-06-15

**Authors:** Francesca Felicia Operto, Grazia Maria Giovanna Pastorino, Andrea Viggiano, Giovanni Battista Dell’Isola, Gianluca Dini, Alberto Verrotti, Giangennaro Coppola

**Affiliations:** 1 Child and Adolescent Neuropsychiatry Unit, Department of Medicine, Surgery and Dentistry, University of Salerno, Baronissi, SA, Italy;; 2 Department of Medicine, Surgery and Dentistry “Scuola Medica Salernitana”, University of Salerno, Baronissi, SA, Italy;; 3 Department of Pediatrics, University of Perugia, Giorgio Menghini Square, 06129 Perugia, Italy

**Keywords:** Epilepsy, cognitive impairment, intellectual disability, anti-seizure medication, ketogenic diet, ganaxolone, gene therapy

## Abstract

Managing epilepsy in people with an intellectual disability remains a therapeutic challenge and must take into account additional issues such as diagnostic difficulties and frequent drug resistance. Advances in genomic technologies improved our understanding of epilepsy and raised the possibility to develop patients-tailored treatments acting on the key molecular mechanisms involved in the development of the disease. In addition to conventional antiseizure medications (ASMs), ketogenic diet, hormone therapy and epilepsy surgery play an important role, especially in cases of drugresistance. This review aims to provide a comprehensive overview of the mainfactors influencing cognition in children and adolescents with epilepsy and the main therapeutic options available for the epilepsies associated with intellectual disability.

## 1 INTRODUCTION

Epilepsy is one of the most common neurological disorders, affecting over 70 million people worldwide [1]. The prevalence of epilepsy is higher in people with intellectual disability (ID) than in the general population and seems to increase with the severity of disability [2]. Early-onset, polytherapy, symptomatic etiology and poor seizure control are considered risk factors for neuropsychological deficit [3-6]. Epilepsy and cognitive disorders are strongly associated with epileptic encephalopathies, where the epileptic activity itself contributes to the genesis of severe intellectual or behavioral disabilities [7]. Indeed, a long history of seizures and multiple episodes of status epilepticus can cause progressive changes in brain connectivity and lead to cognitive decline over time [8]. In addition, interictal epileptiform discharges can result in transitory cognitive impairment [9-11]. Thus, achieving good seizure control becomes a fundamental goal, aimed at improving the quality of life of patients and caregivers. Beside conventional antiseizure medications (ASMs), new drugs and non-pharmacological approaches play an important role, especially in cases of drug resistance and drug toxicity. This review aims to provide a comprehensive overview of the main factors influencing cognition in children and adolescents with epilepsy and the main therapeutic options available for the epilepsies associated with intellectual disability.

## 2 MAIN FACTORS INFLUENCING COGNITION IN EPILEPTIC CHILDREN AND ADOLESCENTS

It is important to underline that global intelligence is the result of multiple, complex and interconnected cognitive domains.

The standardized tests of intelligence most used in clinical and research fields are the Wechsler Intelligence Scales, which provide a Total Intellectual Quotient (TIQ) from the integration of the different cognitive sub-domains: verbal comprehension, which is the ability to understand, process and use acquired verbal information; visual-spatial reasoning, which represents non-verbal logical reasoning skills; working memory, which is the ability to retain information in the short term memory in order to perform cognitive tasks; the processing speed, which is the rapidity of execution of visuo-spatial and visuo-motor tasks.

Each of these cognitive abilities can be compromised in subjects with epilepsy, with particular involvement of executive functions (*e.g*. working memory, inhibition, flexibility, planning and problem solving) and processing speed. Other cognitive functions that can be impaired can be visuo-spatial memory, social cognition and learning skills.

On the basis of the standardized evaluation of the TIQ, we can identify the following conditions: normal intelligence TIQ>84; borderline TIQ = 70-84; mild intellectual disability TIQ = 50/55 - 69; moderate intellectual disability TIQ = 50/55 - 35/40; severe intellectual disability TIQ = 35/40-20/ 25; profound intellectual disability TIQ <20/25.

Cognitive disorders in children with epilepsy are common, ranging from mild to profound intellectual disability and covering distinct cognitive domains.

All this strongly depends, on the age of onset, the etiology of epilepsy and the type of epileptic syndrome.

In a significant study by Berg *et al.* (2008) [12] conducted on over 600 children with newly diagnosed epilepsy and followed for a median of 10.5 years, the cognitive level was considered normal in 73.6% of cases, while a TIQ <80 was reported in 26% (borderline in 5.1%, mild in 3.4%, moderate in 7.3%, severe delay in 4.7% and impaired but not further classified in 5.9%). Factors strongly associated with cognitive functioning were age at seizure onset (<5 years), symptomatic etiology, epileptic encephalopathy, duration of seizure remission and type of anti-seizure treatment. Multivariable logistic regression analysis showed that all these factors, except the state of remission, independently contribute to the global cognitive functioning below the average.

In another study by Reilly *et al.* (2015) [13] which assessed the cognitive level in school-aged children with active epilepsy, approximately 24% had a TIQ <50 and 40% had a TIQ below 70. Furthermore, Schouten *et al.* (2001) [14] reported that 20% of children with epilepsy repeat at least one year at school and about 50% require a supportive teacher before the diagnosis of epilepsy is made. These authors conclude that a cognitive disorder is not limited to children with refractory epilepsy, but also to those with generalized or focal genetic epilepsies such as rolandic seizures.

In agreement with these data, cognitive and structural abnormalities associated with Benign Epilepsy with Centro-Temporal Spikes (BECTS) have been confirmed by neuroimaging studies, which have shown an atypical maturation of the cerebral cortical thickness in the frontal and parietal-occipital region prior to the onset of idiopathic focal seizures [15, 16]. Furthermore, these anomalies tend to evolve over time, even if cognitive functions appear more resistant to further changes than anatomical anomalies.

The ultimate cognitive functioning and the developmental outcome of children and adolescents with epilepsy appear to depend on a complex interaction between multiple factors, as illustrated in Fig. (**1**). As it can be seen, the developmental potential of the child is primarily determined by the genetic background (*e.g*. the parental IQ), and the environmental background (educational level of the parents, socio-environmental and socio-cultural factors within and outside the family).

### 2.1 Intellectual Functioning and Genetic Background

In a study by Hermann *et al.* (2016) [17], the neurophysiological and clinical patterns and the quantitative magnetic resonance data were evaluated in 138 children between 8 and 18 years of age with newly diagnosed idiopathic epilepsies and in 95 controls. The confirmatory factor analysis allowed us to identify 5 cognitive factors (verbal, perceptual, speed, attention, and executive), which led to the distinction of 3 clusters of patients: 1) average and comparable with controls, 2) mild impairment across multiple cognitive domains, and 3) impairment across all domains with severe attentional impairment, representing 44%, 44% and 12% of the epilepsy sample, respectively.

The cognitive profile of these 3 groups was associated with increasing anomalies in the brain structure, with the Intelligence Quotient (IQ) of the parents and with the characteristics of the early psychomotor development, and not with the epileptic syndrome. The same study methodology had previously been applied to patients with chronic temporal lobe epilepsy (TLE), whose modal cognitive profile was characterized by worse performance in all the analyzed domains (attention, executive, perceptual, speed and verbal). These profiles, at a further latent class analysis, were distinct into 3 subgroups: the first with neuropsychological functions comparable to the controls, a second with worsening of memory/executive functions and the third with a global cognitive impairment, in particular relating to the executive function and the processing speed.

Similarly to what was found in TLEs, in patients with idiopathic epilepsies, there were minimal brain structural abnormalities in the first group and more significant changes in the third group, as compared to controls. These anomalies consisted of a smaller volume of the subcortical gray matter and of the cortex of both cerebellar hemispheres, as well as a smaller volume of the thalamus bilaterally. These subgroups (clusters 1-3) were associated with characteristics of the family environment, the early stages of psychomotor development and the age at epilepsy onset.

In particular, the children of the third group had parents with a lower IQ (though within normal limits), a greater need for language and neuropsychomotor treatment before the age of 3 years and an earlier onset of epilepsy. The authors conclude that distinct cognitive profiles can be identified in childhood idiopathic epilepsies, which represent the result of a wide range of risk factors, including parental genetic background. In agreement with this are the data coming by Meekes *et al.* (2015) [18], who tested the hypothesis that a change in the IQ of children undergoing surgery for epilepsy is associated with the educational level of their parents. The retrospective analysis of a cohort of 118 children (60 male, median age at surgery of 9.7 years) found that parental education level was associated with the delta IQ at simple regression analysis (p = 0.004), also contributing to post-surgical IQ, as it was disclosed by multiple regression analysis (p = 0.008).

In other words, children whose parents had a higher level of education demonstrated on average, a greater increase in IQ after surgery and a higher post-surgical (but not presurgical) IQ than children whose parents had completed a lower secondary school cycle. As a consequence, the authors state that parental education level, together with other potential environmental factors, should be considered for functional outcomes after surgical treatment of childhood epilepsy. In fact, parental education level is not only an environmental factor, positively influencing the cognitive post-surgical recovery of a child within his/her family as well as a factor linked to more or less economic resources for cognitive rehabilitation, but it is also a factor potentially correlated with parental IQ, which represents a genetic component to be taken into account.

Family factors, including parental education, household income, family mastery, social support and family demands, were somewhat less significant in children and adolescents with drug-resistant epilepsy, aged between 4 and 18 years, evaluated for possible surgical treatment of epilepsy [18]. Univariance analysis showed that a higher IQ was associated with older age at epilepsy onset, fewer ASMs, a shorter duration of epilepsy, and unilobar epileptogenic foci. Thus, familial factors globally showed a lower impact on children's IQ than the more typical clinical features of drug-resistant epilepsy. However, Oostrom *et al.*, (2003) [20] confirmed that early cognitive and behavioural problems can be found in children with idiopathic or cryptogenic epilepsies just before the onset of pharmacological treatment, thus being linked to pre-existing factors and not only to epilepsy itself. In 51 outpatient school children with idiopathic or cryptogenic epilepsies and in 48 sex-matched classmate control subjects, these authors conducted a follow-up that included a neuropsychological evaluation repeated three times during the first year after diagnosis, associated with questionnaires administered to parents and teachers for the behavioural aspect. Basically, children with epilepsy showed worse scores on cognitive-behavioural tests already at baseline and before the start of therapy, maintaining this profile also during the follow-up. The authors conclude that contextual factors are all relevant in the very early stages of the disease.

### 2.2 Other Factors Related to the Epileptic Disorder

More specific factors relating to the epileptic disorder must then be considered, such as the underlying epileptic pathology, which mainly includes structural, genetic, metabolic and inflammatory alterations, the possible presence of epileptic encephalopathy, the burden of frequent seizures and epileptic states, the entity of interictal epileptic EEG discharges, the use of anti-seizure drugs and the possible surgical treatment of focal epilepsies. All these factors, isolated or more often connected to each other, can induce structural and / or functional alterations of brain connectivity and of more complex networks, eventually influencing cognitive functions and neurodevelopment.

As for the epileptogenic pathology with its multiple causes, one can think of preventing or blocking cognitive deterioration through early identification and targeted treatment of the underlying progressive pathology, such as immunomodulation for autoimmune encephalitis and other inflammatory disorders or through dietary supplements or pharmacological therapies for the metabolic causes of epilepsies [21-23].

### 2.3 Neurodevelopment and Gene Mutations

Disorders of cognitive functions up to intellectual disability and/or other neurodevelopmental disorders such as autism associated with the development of epilepsy may be the result of genetic conditions that involve the same pathophysiological mechanisms, that is, a disorder of synaptic plasticity. The latter leads to an imbalance of excitation and inhibition in the developing brain, as can be found, for example, in disorders like Fragile X-syndrome, CDKL5 encephalopathy, and tuberous sclerosis complex (TSC), neuroligin mutations, interneuronopathies (*e.g*., ARX) and NRP2 mutations [24]. Abnormalities in synaptic plasticity can result from alterations in receptors, molecular signals or neurotrophins, many of which are associated with early seizures. Mutations in genes implicated in neurodevelopment associated with epilepsy can lead to multiple functional alterations involving enzyme/enzyme modulation, receptor transporter/receptor transporter, cell adhesion molecules, extracellular matrix, membrane structures, membrane trafficking, proteins of the cytoskeleton, the binding of nucleic acids alongside a group of unclassifiable. The largest groups concern enzyme/ enzyme modulation and cytoskeletal proteins.

Genome-wide studies in patients with epilepsy and neurodevelopmental disorders, including intellectual disability and/or autism spectrum disorder, showed copy number variations (CNVs) such as 2q24.2-q24.3, 7q11.22, 15q 11.2-q13.3 and 16p13.11-p13.2, some of which alter multiple genes like NRXN1, AUTS2, NLGN1, CNTNAP2, GIN2A, PRRT2, NIPA2 and BMP5 [25]. In addition, recent exome sequencing studies have shown mutations in genes of non-ionic channels such as LGI1, PRRT2, EFC1, PRICKLE, RBFOX1 and DEPDC5, also implicated in neurodevelopment [25, 26].

Conditions associated with intellectual disability, epilepsy and/or autism spectrum disorder include as well cortical developmental malformations (CDM), which constitute a broad spectrum of focal, regional or diffuse structural brain abnormalities. CDMs, as the acronym suggests, consist of perturbations of the neuronal cytoskeleton, that is, of the normal architecture of the cerebral cortex and hippocampus. The pathogenesis of these disorders is certainly complex, and an important factor is represented by an altered radial or axial neuronal migration, largely based on mutations of genes that regulate the migration of newly born post-mitotic neurons. The genes involved mainly code for proteins involved in the function of the cytoskeleton as well as in cell division and in the formation of axons/dendrites [27]. Phenotypes that relate to CDMs genes largely include intellectual disability and epilepsy/seizures and are characterized by lissencephaly (*e.g*., DCX, LIS1, ARX, TUBA1A, RELN, FLNA genes), lissencephaly type 2/micropolygyria (especially TUBB2B, GPR56, SRPX2), microcephaly (ASPM, MCPH1, STIL, GENPJ, WDR62, KIF5C) [27].

The important role of epigenetic factors in neuronal functioning from embryogenesis and early brain development up to the genetic expression of specific tissues should also be considered. Epigenetic factors indeed modulate the gene-environment interaction, thus influencing neurodevelopmental disorders and epilepsy. Common epigenetic mechanisms include DNA methylation, post-translational histone modification, and non-coding RNA [28]. These factors determine not only gene expression or its silencing, but also how and when a gene is expressed and which proteins are transcribed. All these factors are also influenced by age and environmental factors such as nutrition, smoking, drugs and chemicals, as well as psychosocial and emotional events such as mental stress.

Among the non-coding RNA factors, there are also the micro-RNAs that modulate the translation of target genes in the early stages of brain development, playing a critical role in the purposes of dendritogenesis, synaptic formation and maturation.

Let us now review some well-established or more recently reported disorders due to CNVs or gene mutations associated with intellectual disability and epilepsy [29].

The chromosomal 15q11-q13 regions are structurally complex, and their abnormalities are associated with various neuropsychiatric disorders, including autism spectrum disorder, epilepsy, Angelman syndrome, and Prader-Willi syndrome; so children with intractable epilepsy, Autism Spectrum Disorder (ASD), and language and motor retardation should be considered to have this syndrome [30].

ST3GAL3 deficiency is a rare autosomal recessive disorder caused by pathogenic mutations in the ST3GAL3 gene and characterized by epilepsy, motor development delay, severe intellectual disability and behavioral disorders [31].

Cardio-facio-cutaneous (CFC) syndrome is an extremely rare autosomal dominant genetic disease due to BRAF and other gene mutations. The main characteristics of these patients are craniofacial deformities, cardiac malformations, skin abnormalities, delay of language and motor development, gastrointestinal dysfunction, intellectual disability, and epilepsy. A recent study has reported the case of a child with a typical CFC syndrome, developmental delay and a de novo heterozygous mutation c.1741A>G (p. Asn581Asp) in exon 14 of the BRAF gene [32].

Nicolaides-Baraitser syndrome is caused by a mutation in the SMARCA2 gene that goes along with intellectual disability, congenital malformations of the face and limbs and often difficult-to-treat epilepsy [33].

Recently authors have reported a patient with a novel autosomal recessive pathogenic variant in SPATA5 and a clinical phenotype consistent with SPATA5 syndrome, including severe neurological impairment, intellectual disability, generalized intractable epilepsy, microcephaly, abnormal muscle tone, and sensorineural hearing loss [34].

Interstitial duplications of 3q29 have recently been described in association with a new genetic syndrome characterized by a neurodevelopmental phenotype. A total of 16 individuals with the 3q29 duplication have been reported in the literature with clinical features that include intellectual disability, language delay, epilepsy, structural brain anomalies, micro/macrocephaly, generalized obesity, ocular abnormalities, distinctive facial features, cleft palate, and musculoskeletal anomalies. Recently, eleven additional cases from nine families with the 3q29 microduplication have been identified by microarray analysis [35].

Pathogenic variants in SCN2A are reported in a spectrum of neurodevelopmental disorders, including developmental and epileptic encephalopathies, benign familial neonatal-infantile seizures, episodic ataxia, and autism spectrum disorder and intellectual disability with and without seizures. To date, more than 300 patients with SCN2A variants have been published, the majority presenting with epilepsy [36].

Chromosome 14q11-q22 deletion syndrome is a rare contiguous gene syndrome. Two regions of overlap (RO) of the 14q12q21.1 deletion have been identified: a proximal region (RO1), including FOXG1(*164874), NKX2-1(*600635), and PAX9(*167416) and a distal region (RO2), NKX2-1 and PAX9. A recent study reports a new RO, not including the previously reported candidate genes that encompass the distal breakpoint of RO1 and the proximal breakpoint of RO2, and seems to be associated with intellectual disability, hypotonia, epilepsy, and corpus callosum abnormalities, in a 6-year-old boy with mild dysmorphic facial features, global developmental delay, and hypoplasia of the corpus callosum [37].

Seizure threshold 2 (SZT2) gene mutations have been associated with developmental and epileptic encephalopathies (DEEs). The main clinical features related to SZT2 variants are epilepsy with onset within the first years of life, intellectual disability, macrocephaly with dysmorphic facial features, corpus callosum (CC) shape abnormalities and cortical migration disorders. The c.7825T>G homozygous missense variant in SZT2 has been identified in two female siblings presenting with focal seizures, mild-moderate ID, behavioral disturbances, and facial dysmorphisms [38].

Pathogenic variants in SCN2A are reported in a spectrum of neurodevelopmental disorders including developmental and epileptic encephalopathies, benign familial neonatal-infantile seizures, episodic ataxia, and autism spectrum disorder and intellectual disability with and without seizures [36].

Recently, genetic variation within the GRIN2D gene, which encodes the GluN2D subunit of the NMDAR, has been associated with a set of early-onset neurological diseases, notably developmental and epileptic encephalopathy (DEE) [39].

A new case of a boy with severe intellectual disability with absent speech, autistic spectrum disorder, mild dysmorphic facial features, failure to thrive and epilepsy has been described in literature, having a de novo heterozygous missense mutation in EEF1A2 (c.364G>A; p.Glu122Lys) identified by next generation sequencing. Most clinical features are shared by all individuals with EEF1A2 mutation, but this patient showed a mild epileptic phenotype that developed in late infancy and was well-controlled with antiepileptic drugs [40].

SCN3A is a recently recognized gene associated with neurodevelopmental disorder and epilepsy [41].

PCDH19-Girls Clustering Epilepsy (GCE) is an epileptic syndrome with infantile-onset, characterized by clustered and fever-induced seizures, often associated with intellectual disability and autistic features [42].

Heterozygous loss of function variants in the IRF2BPL is a newly described cause of neurodevelopmental disabilities and epilepsy [43].

De novo STXBP1 mutations are among the most frequent causes of epilepsy and encephalopathy. Most patients have severe to profound ID with little correlation between seizure onset, seizure severity, and the degree of ID [44].

Mutations in tubulin genes are responsible for a large spectrum of brain malformations secondary to abnormal neuronal migration, organization, differentiation and axon guidance and maintenance. Motor impairment, intellectual disability and epilepsy are the main clinical symptoms [45].

Pathologic mutations in cyclin-dependent kinase-like 5 cause CDKL5 deficiency disorder, a genetic syndrome associated with severe epilepsy and cognitive, motor, visual, and autonomic disturbances. This disorder is a relatively common genetic cause of early-life epilepsy [46].

POGZ encodes a multidomain nuclear protein involved in transcriptional regulation and its defective function has been recently associated with a syndromic neurodevelopmental disorder, known as White-Sutton syndrome. While originally epileptic seizures were unreported, it seems that epilepsy represents a recurrent feature in affected subjects. Few data, however, are available on electroclinical features of POGZ-related epilepsy. A recent study reported a 5-year-old girl with a de novo inactivating POGZ mutation with a complex neurological phenotype characterized by hypotonia, severe developmental delay, and paroxysmal epileptic and nonepileptic events [47].

20p13 microdeletion syndrome has been reported to be associated with developmental delays, intellectual disability, epilepsy, and unspecific dysmorphic characteristics. However, only a few cases of 20p13 microdeletion have been described, and therefore its typical features and precise pathogenesis remain elusive [48].

PUM1 has been very recently reported as responsible for a new form of a developmental disorder named PADDAS syndrome. A recent study describes a patient with early-onset developmental delay, epilepsy, microcephaly, and hair dysplasia, with a de novo heterozygous missense variant of PUM1: c.3439C > T, p.(Arg1147Trp) [49].

De novo mutations in PURA have recently been described to cause PURA syndrome, a neurodevelopmental disorder characterized by severe intellectual disability, epilepsy, feeding difficulties and neonatal hypotonia [50].

Mutations in MED17 may define a phenotype characterized by progressive microcephaly, intellectual disability, seizures, cerebellar atrophy of variable degree, and ataxia. Recently the case of two siblings presenting with failure to thrive in early years, progressive microcephaly, moderate intellectual disability, developmental delay, ataxic gait and seizures with an identical EEG pattern, and minimal cerebellar atrophy has been reported. Authors recommend testing MED17 mutations in any patient presenting with two or more of the aforementioned signs and symptoms [51]. In Table **1** are summarized the principal gene mutations covered in this paragraph.

### 2.4 Epileptic Encephalopathy: New Concept

The epileptic disorder may, especially in childhood, be underlying an epileptic encephalopathy which is another target to try to prevent or slow down cognitive impairment in developing brains. First, we must recall the new concept of epileptic encephalopathy as a condition in which the epileptic anomalies are hypothesized to be themselves responsible for the deterioration and progressive worsening of cognitive function. Nonetheless, there is still a debate among those who consider that the underlying etiology is more relevant and that reducing or blocking epileptic activity has a limited impact on cognitive prognosis, and those who believe that optimizing seizure control and reducing epileptogenic discharges may result in a cognitive improvement [52].

In other words, there are children in whom there is a clear temporal correlation between cognitive decline and/or psychomotor development and increased seizures and epileptic EEG activity, so that cognitive abilities will benefit from better control of epilepsy, regardless of the etiology [53]. Consistent with this concept, there are at least eight studies confirming that an interval from spasm onset to therapy initiation <4 weeks is prognostically better than an interval >4 weeks for neurodevelopmental prognosis (LTTT ratio 1.519; 95% CI: 1.064-2.169) [54]. Similarly, in the case of Epilepsy with continuous spikes and waves during slow sleep (CSWS), a longer duration is associated with worse verbal and performance IQs after the disappearance of the CSWS [55]. Another important factor linked to cognitive functions in children with epilepsy is the “load” of the seizures which therefore represents a target for the prevention and treatment of cognitive disorders. Several authors agree, in this regard, that cognitive outcome is mainly linked to drug resistance, to an early onset of seizures, and to chronic epilepsy [56].

To confirm these data, there are functional neuroimaging studies that report that human epilepsy is associated with changes in volume in specific brain areas [57], in the structure of the white matter [58] and alterations in the functional and structural characteristics of the entire brain system [59]. Recent studies that evaluated functional and structural networks in terms of “graph theory” in epilepsy, have demonstrated specific alterations in the connectivity and topology of the networks and, consequently, have shifted the attention from the “foci” to the “networks” as regards the research in epilepsy.

In patients with chronic epilepsy, the alteration of these networks can be associated with cognitive and behavioral disorders. According to Diessen *et al.* (2013) [58], as the disease progresses, the topology of the networks becomes less efficient than in healthy controls; potential modifiers of this effect are anti-seizure medications, structural lesions and / or seizure frequency.

Functional neuroimaging studies in animal models of focal epilepsy seem to confirm this data [60]. In the brains of these animals, the interhemispheric functional connectivity was decreased while the intrahemispheric one was increased. Early initiation of therapy and optimal seizure control can, at least in theory, prevent the progressive alterations of the white matter and the brain networks induced by seizures and thus reduce the risk of cognitive decline in children with epilepsy.

### 2.5 Interictal EEG Discharges and Cognitive Function

As for focal and generalized interictal epileptiform discharges, and their role in contributing to cognitive impairment, including various neuropsychological functions, this is an “old” and controversial topic, especially in relation to the question of whether to treat these discharges in the absence of clinical seizures.

As demonstrated by Binnie (2003) [61] in his historical studies, even a single focal or generalized interictal discharge can be associated with a transient cognitive impairment (TCI). On the contrary, there is a much lower agreement on the real impact of interictal epileptic discharges in the long term on cognitive functioning. According to Fernandez *et al.* (2015) [62], there is currently no evidence either for or against treating epileptic discharges. Interictal discharges should only be treated if there is a well-demonstrated close correlation between cognitive regression and paroxysmal EEG abnormalities, even in the absence of clinical seizures.

## 3 TREATMENT OPTIONS AVAILABLE FOR THE EPILEPSIES ASSOCIATED WITH INTELLECTUAL DISABILITY

### 3.1 Introduction

The treatment of epilepsy in patients with ID follows the same principles valid for the general population and is aimed to achieve complete seizure control without incurring severe adverse effects (AE). However, managing epilepsy in people with ID must take into account some additional issues, such as communication difficulties, neuropsychiatric comorbidities, frequent drug resistance and a greater risk of AE [63]. Moreover, patients with both epilepsy and ID are often treated with multiple types of drugs, such as antipsychotics and anti-depressants, with an additional impact on cognitive performance.

### 3.2 The Role of Antiseizure Medications

Although ASMs share the purpose of controlling seizures, they act with different mechanisms, including the modulation of voltage-gated ion channels, enhancement of GABA-mediated inhibition and the blockade of NMDA receptors. Many of them exert different types of action, while the mechanisms of others are still unknown [64].

The choice of a specific drug is based on the seizure type, epilepsy syndrome, patient's comorbidities, and potential AE and drug interactions [65]. In general, it is preferable to use broad-spectrum ASMs, possibly in monotherapy and giving preference to non-sedative drugs.

Valproic acid (VPA), lamotrigine (LTG) and topiramate (TPM) are considered safe and effective drugs in patients with both epilepsy and ID, while phenytoin and phenobarbital are generally avoided for their sedative effects [66-69]. Some drugs find specific indications in certain syndromes.Vigabatrin is the first-line therapy for West syndrome related to tuberous sclerosis complex [70]. Carbamazepine and lacosamide can have a favorable effect on behavior and attention in children with epilepsy-Attention Deficit-Hyperactivity Disorder (ADHD) comorbidity [71]. By contrast, LTG can aggravate seizures in Dravet Syndrome (DS) patients [72].

Since ASMs act by modulating neuronal activity, it is not surprising that many of them can negatively affect cognition and behavior. For example, TPM and zonisamide, can have a negative impact on executive function [73], while levetiracetam and perampanel do not seem to impair cognitive functional though they can lead to behavioral side-effects such as aggression and irritability [16, 74, 75]. These effects are often dose-dependent and can be minimized by gradual dose titration [76, 77]. Rufinamide does not appear to lead to a deterioration of the cognitive profile in patients with Lennox-Gastaut [78]. However, in patients with ID, it is often difficult to determine whether the cognitive effects are attributable to the ASMs or to the patient's underlying condition, comorbidities or recurrent seizures. In some cases, the secondary effects of ASMs can also be beneficial and improve psychiatric comorbidities. VPA and LTG showed mood-stabilizing properties, while gabapentin and pregabalin have an anxiolytic effect [79]. Weight loss caused by TPM [80], contrary to weight gain induced by other ASMs (*e.g*., VPA), may be considered an optimal effect in patients with ID in whom obesity is more common [81]. In addition to conventional ASMs, newer agents, including fenfluramine and cannabidiol, showed efficacy as adjunctive treatments in DS and may have a positive effect on cognitive functioning in these patients [82].

### 3.3 Precision Medicine

Despite numerous advances in the diagnosis and treatment of epilepsy, almost 30% of patients still do not respond to conventional therapy [83, 84]. Hence the need to develop new drugs acting on the key molecular mechanisms involved in the development of the disease. This individually tailored, molecular-based treatment approach is called precision medicine (Table **2**). Precision medicine represents a great challenge because the etiological basis of epilepsy is complex and not fully understood. A gene mutation (*e.g*., SCN1A) can be expressed in a broad spectrum of clinical phenotypes [85] and vice versa; the same clinical condition can be caused by different genetic mechanisms.

An example of precision medicine is represented by the use of the antagonists of the NMDA receptor (NMDAR), such as memantine, in epileptic disorders related to the GRIN2A gene mutation. In particular, the GRIN2A missense mutation (c.2434C>A; p.L812M) leads to increased activity of the NMDAR and promotes epileptic activity. Based on these data, memantine was used as adjunctive therapy in a 9-year-old patient with the GluN2A-L812M mutation and a clinical history of ID and intractable seizures, achieving a reduction in seizure frequency and improvement in EEG recording [86]. Mir *et al.* described a patient with a novel GRIN2A mutation and severe drug-resistant epileptic spasms who became seizure-free after treatment with memantine [87].

The use of quinidine for the treatment of KCNT1-related epilepsy represents another case of precision medicine. Quinidine inhibits *in vitro* activity of KCNT1 channels [88], hence the hypothesis that this drug may represent a targeted therapy for epilepsies related to KCNT1 mutations [89]. Gain-of-function mutations in the potassium channel gene KCNT1 are associated with various epileptic disorders, including severe autosomal dominant nocturnal frontal lobe epilepsy (ADNFLE) and epilepsy of infancy with migrating focal seizures (EIMFS) [90, 91]. Milligan *et al.* reported the use of quinidine in two patients. One, carrying the Tyr796His mutation, presented severe nocturnal focal and secondary generalized seizures and did not achieve clinical improvement after treatment. The second patient was a case of EIMFS caused by Lys629Asn mutation. In this patient, quinidine therapy led to an 80% reduction in seizure frequency [88].

Precision medicine can also be applied for the targeting of loss-of-function mutations, as in the case of epilepsies related to mutations of the KCNQ2 gene, encoding the voltage-gated potassium channel Kv7.2. Loss-of-function mutations of KCNQ2 can express in a wide range of neurological disorders ranging from benign neonatal epilepsy to severe epileptic encephalopathy [92]. Retigabine acts as a positive allosteric modulator on Kv7.2 [93] and has shown to reduce the frequency of seizures in patients with neonatal-onset epileptic encephalopathy carrying KCNQ2 mutation [94].

### 3.4 Gene Therapy

Several forms of epilepsy associated with ID present recurrent gene mutations. Recent developments in next-generation sequencing technics have led to the identification of more than 100 genes underlying this condition [99]. Involved genes can be grouped into at least 3 categories: i) gene affecting the regulation of other genes, ii) gene affecting neuronal excitability, iii) gene affecting synaptic transmission [100]. Whether structural abnormalities or altered synaptic transmission are the underlying pathogenic mechanism remains unknown. However, in both cases, a prompt and adequate therapy through ASMs or surgery often does not lead to a resolution of symptoms. Hence the need to improve new targeted therapies for specific gene mutations. The primary goal of gene therapy is to modulate the altered gene expression. This requires both a precise knowledge of gene mutations and altered pathways underlying the disease, as well as an approach that allows a targeted effect. Transgene supplementation is one of the most studied approaches followed by antisense oligonucleotides (ASO) that reduce targeted genes expression [101, 102]. A more sophisticated technique is genome editing which aims to modify or replace with great precision small parts of the DNA sequence. The most recent technologies of genome editing are three: the Talen nuclease, the Zn-finger nuclease and the Cas9 nuclease (*i.e.*, The Clustered Regularly Interspaced Short Palindromic Repeat CRISPR system). The most commonly used delivery vectors are adeno-associated virus (AAV) vectors [103]. In particular, AAV9 represents the most used choice in neurological disorders for its high neurotropism and ability to cross the blood-brain barrier [104]. The systemic or stereotactic administration can be chosen considering in the first case the greater convenience and in the second case the greater effectiveness.

To date, mainly pre-clinical studies have evaluated gene therapy on epilepsy-associated with ID, reporting promising results [105]. An improvement in survival and phenotypic severity in mecp2-null male mice was described after mecp2 gene replacement through AAV [106-110]. Although gene therapy has resulted in beneficial effects, further studies are needed to evaluate the severe side effects described and to assess the possible use of gene therapy in patients with Rett syndrome [111].

Gene therapy has also led to an improvement in specific behavioral abnormalities and restored hippocampal synaptic function in the mouse model of fragile X syndrome (FXS) [112, 113]. The CRISPR system was also applied to FXS resulting in the reactivation of FMR1 mRNA expression [114-116].

Targeted genomic integration with AAV vectors can restore patUBE3A in Angelman syndrome [117]. Meng *et al.* reached the same results using ASO and rescued contextual fear learning, without behavioral and motor improvement [118].

Intravenous and intraventricolous injection of an AAV9 vector encoding hamartin in tuberous sclerosis mouse models resulted in extended lifespan and a marked reduction in brain pathology [119, 120].

A large proportion of genetically based epilepsies associated with ID are caused by mutations in ion channels. These forms, such as DS, present important limitations to gene therapy primarily because of the large size of the malfunctioning ion channels exceeding the capacity for AAVs [121]. Targeted augmentation of nuclear gene output (TANGO) technology has been applied to DS, resulting in increased levels of productive mRNA and sodium channels [122]. Intracerebroventricular administration of an ASO (STK-001) in mice models led to a global reduction in seizures and Sudden Unexpected Death in Epilepsy (SUDEP) [123, 124]. STK-001 has also been tested on non-human primates confirming good tolerability and outcomes [125]. These results led to the initiation of a clinical trial studying the STK-001 administration in children and adolescents with ds with promising results [126]. However, it remains to limit the use of this technique only for mutation with haploinsufficiency. Other techniques, such as CRISPR-Cas9/AAV strategies, have been applied to DS only in preclinical studies [127, 128].

Gene therapy certainly represents a promising therapy with curative and not only symptomatic potential. However, there are still several limitations that affect the use of these techniques. Further studies and clinical trials are needed to investigate gene therapy in patients with epilepsy and ID.

### 3.5 Hormone Therapy

Hormones and epilepsy are linked by a complex interaction [129]. Indeed, hormones modulate neuronal excitability resulting in an anticonvulsant or proconvulsant effect [130-132]. On the other hand, seizures may alter the release of hypothalamic and pituitary hormones [133]. Thus, various hormones or derivatives, including ACTH, corticosteroids and ganaxolone (GNX), are used for the treatment of epilepsy.

GNX (3α-hydroxy-3β-methyl-5α-pregnan-20-one) is a synthetic neurosteroid acting as a positive allosteric modulator of the GABAA receptor [134]. Several studies assessed the safety and efficacy of GNX in patients with epilepsy and intellectual disability. GNX has proven effective in reducing the frequency of seizures in cases of infantile spasms [135], PCDH-19 related epilepsy [136], LGS [136] and CDKL5 deficiency disorder [137].

The efficacy of ACTH in infantile spasms was firstly reported in 1958 [138]. Since then, corticosteroids have been used for many other epileptic disorders, including LGS and Landau-Kleffner Syndrome [139-142]. Two different preparations of ACTH are available: a natural ACTH of bovine derivation and a synthetic analogue called tetracosactide [143]. The antiepileptic mechanism of ACTH is not fully understood. It can probably reduce neuronal excitability both by inducing steroid release and by direct action on the melanocortin receptors [144]. For the treatment of West syndrome, the FDA recommends an ACTH dose of 150 IU/m2/day (divided into twice daily intramuscular injections of 75 IU/m2) administered over a 2-week period and gradually tapered to avoid adrenal insufficiency. However, several studies have compared the efficacy between high-dose and low-dose ACTH, without showing significant differences [145-147].

Among steroids, the most used in epilepsy are prednisolone and hydrocortisone [148].

### 3.6 Ketogenic Diet

A ketogenic diet (KD) is a high-fat and low-carbohydrate diet first introduced in epilepsy treatment in the 1920s [149]. The classic KD provides a ketogenic ratio (grams of fat:carbohydrate and protein combined) of 4:1, in order to determine a constant state of ketosis. Systemic ketosis forces brain tissue to switch from consuming glucose to consuming ketone bodies as energy substrates. This metabolic modification has a marked antiepileptic effect, as demonstrated in animal models and humans [150-152]. Numerous mechanisms of action have been proposed to explain the anticonvulsant effect of KD, including the increase of GABA synthesis, a decrease of adenosine and norepinephrine levels, the enhancement of mitochondrial function and oxidative stress reduction [153]. However, the exact underlying mechanism of the KD remains unclear [154].

KD presents some difficulties of application which mainly concern compliance and AE. The most common AE include gastrointestinal disturbances, growth arrest, hypercholesterolemia, kidney stones and, in some cases, severe episodes of ketoacidosis [155].

In addition to the classic KD, there are alternative regimens which include the medium-chain triglyceride diet (MCTD), the modified Atkins diet (MAD) and the low glycemic index treatment (LGIT) [156]. While the classical KD mainly consists of long-chain triglycerides (LCTs), the MCT diet involves the intake of large quantities of medium-chain triglycerides (MCTs).

The advantage of MCTs is linked to the higher production of ketone bodies per kilocalorie of energy compared to LCTs. Therefore, the MCT diet requires less fat intake to achieve ketosis.

MAD provides an approximate 1:1-2:1 ketogenic ratio, without specification on which types of carbohydrates can be eaten [157, 158]. Finally, LGIT is a less restrictive diet that emphasizes both the total amount and the type of carbohydrates introduced, allowing only those with a Glycemic Index <50 [159].

KD is the treatment of choice for glucose transporter type 1 (GLUT1) deficiency syndrome (GLUT1-DS) [160], a neurometabolic disorder characterized by early-onset epilepsy, microcephaly, ataxia, developmental delay and cognitive impairment [161]. KD has proven to be effective in other pharmacoresistant childhood epilepsies, including refractory infantile spasms [161, 162] and Lennox-Gastaut Syndrome (LGS) [163-166].

### 3.7 Immunotherapy

Inflammation may have a key role in the pathogenesis of some forms of epilepsy, and seizure itself can induce the release of proinflammatory mediators in the brain [167]. Hence the concept of an immunological therapy of epilepsy, in which modulation of inflammation can be translated into a favorable effect on seizure control.

Immunological therapy finds a major application in autoimmune encephalitis, neurological disorders mediated by an antibody directed against surface or intracellular neuronal antigens. Seizures associated with autoimmune encephalitis are often refractory to conventional ASMs but exhibit good response to immune-modulating treatments. Moreover, immunologic therapy resulted in a clinical and electroencephalographic improvement even in epileptic syndromes frequently associated with ID that does not recognize a primarily autoimmune etiology [168].

The immunologic therapies mainly used in epilepsy include corticosteroids, plasma exchange, intravenous immunoglobulin (IVIG) and biologic drugs [169].

In particular, oral and intravenous corticosteroids are effective for the short-term treatment of West syndrome [170-172] and showed encouraging results in Landau-Kleffner syndrome [173] and PCDH19-related epilepsy [174].

IVIG is widely used in an increasing number of diseases. Ariizumi *et al.* first reported the clinical efficacy of IVIG in West syndrome [175]. Since then, several studies have evaluated the use of IVIG in various childhood epilepsies syndromes, including LGS, West syndrome, Landau-Kleffner syndrome and the GRIN-related developmental-epileptic encephalopathies [176-178]. However, it is difficult to establish the real efficacy of IVIG in epilepsy due to the heterogeneous conditions of patients treated with IVIG and because most of the studies conducted lack controls.

Among biologics, the main evidences concern rituximab and tocilizumab. Rituximab has demonstrated efficacy as a second-line treatment in autoimmune encephalitis, often in combination with high-dose steroids [179]. The interleukin-6 receptor inhibitor (tocilizumab) is a therapeutic option for autoimmune encephalitis and New-Onset Refractory Status Epilepticus (NORSE) [180] refractory to rituximab [181].

Since autoimmune epilepsies can express not only seizures but also behavioral disorders, they represent a diagnostic challenge, especially in people with ID. An autoimmune etiology must be considered in case of recurrent seizures of unknown origin. If confirmed, early treatment could improve patient outcomes [182].

### 3.8 Surgery

Therapeutic failure in epilepsy, besides negatively affecting morbidity and mortality, has a negative impact on the neurofunctional and psychosocial components [183]. The neurosurgical treatment represents an additional therapeutic option in drug-resistant epilepsy. The primary goal of surgery is to isolate the epileptogenic focus. Main surgical approaches are resections, in which the epileptogenic region is removed, and disconnections, aimed at disconnecting the affected area from the remaining cortical parenchyma. Other palliative approaches are vagus nerve stimulation (VNS), disconnection or removal of the corpus callosum (callosotomy), multiple subpial transections and deep brain stimulation (DBS) [184].

Structural focal epilepsies are the most likely candidates for resections procedure. In fact, it is mandatory to search for focal abnormalities in drug-resistant epilepsy in order to evaluate the indication for surgical treatment. Intraoperative EEG monitoring provides a more accurate delineation of the epileptogenic focus and the surrounding cortical region.

Besides being supported by efficacy data [185], VNS is a technique that can be conducted in an outpatient setting. Often epilepsy associated with ID makes ambulatory surgery less accessible. Mezjan *et al.* demonstrated in a study on 26 patients that VNS is a safe and feasible ambulatory technique also in these patients [186]. Indeed, it results in not only medical but also psychological benefits, improving the difficult management of these patients and reducing the discomfort associated with hospitalization.

Corpus callosotomy resulted more effectively than VNS in atonic seizures and dropped attacks [187].

In the absence of an epileptogenic focus, another surgical approach to consider is DBS. Initially applied only to the anterior nucleus of the thalamus [188], other targets have been later studied, such as the centromedian–parafascicular complex and the hippocampus [189]. Furthermore, there is evidence that DBS can also lead to an improvement in executive function, depression, anxiety, attention, and mood [190]. However, the mechanisms underlying this improvement remain unknown.

In conclusion, particularly in epilepsies associated with the ID in which poor seizure control can lead to a worsening of cognitive level, a prompt surgical evaluation could benefit in case of drug resistance.

### 3.9 Therapeutic/Enabling Interventions for Cognitive Impairment

ID, as well as epilepsy, can be caused by different pathological processes [191]. Impairment of cognitive functions in people with epilepsy can involve global intelligence and individual cognitive functions such as executive functions (*e.g*., working memory), processing speed, visuo-spatial memory, social cognition skills and learning [192-195]. So, cognitive impairment is not a single entity and its treatment requires a multidisciplinary approach to provide the most appropriate and tailored care to the patient. Moreover, the child’s brain has greater plasticity compared to that of adults [196] and starting therapies as early as possible is essential to achieve their maximum effectiveness.

In certain etiologies, presymptomatic treatment may improve the cognitive outcome. In a study conducted by Jóźwiak, infants with tuberous sclerosis complex treated preventively with ASMs, at 24 months of age showed a mean IQ significantly higher (92 versus 69) compared with those treated after the epilepsy onset [197]. Seizures and ID can also result from inborn errors of metabolism [198]. A timely diagnosis and early treatment may limit or even prevent the deleterious consequences on cognitive function typical of these conditions. For example, in GLUT1-DS, the introduction of KD limits both seizures and neurological deterioration by providing alternative sources of energy to the brain (ketone bodies) [199], while in pyridoxine-dependent epilepsy due to ALDH7A1 mutations, treatment with pyridoxine and lysine-restricted diet may improve the neurological outcome [200].

A neuropsychological evaluation is important to identify domain-specific deficits (*e.g*., language, memory, attention, and executive functions), explore possible psychiatric comorbidities such as depression, anxiety, autistic spectrum disorder, and implement appropriate countermeasures [201]. Some ASMs, in addition to controlling seizures, have shown a beneficial effect on the cognitive aspect. For example, LTG has a cognitive-enhancing effect, especially on attention, and some mild improvements on the attentional test were also reported for oxcarbazepine [202]. Choosing ASMs with minimal cognitive side effects, slow titration, avoiding polypharmacy and treating neuropsychiatric comorbidities are important aspects to improve patient’s cognitive outcome. In children with attention deficit hyperactivity disorder (ADHD), methylphenidate may reduce the core symptoms of hyperactivity‐impulsivity and inattention [203]. However, the treatment of patients with epilepsy-ADHD comorbidity is particularly challenging, also due to the effect that some ASMs can have on behaviour [204]. In addition to pharmacological therapies, other types of interventions need to be considered. Physical therapy may improve gross motor skills in people with ID aged 6 years and older [205]. Deep brain stimulation has been used in patients with ID to treat epilepsy, movement disorders and psychopathological disturbances [206-210]. Further studies are needed to evaluate the role of this therapeutic option. Children and adolescents with ID benefit from a comprehensive approach that integrates individualized and interpersonal therapies. Rehabilitation programs, including individualized help in learning new skills, should be implemented. Furthermore, many genetic syndromes associated with both epilepsy and ID have a greater risk of physical comorbidities (endocrine, musculoskeletal, gastrointestinal, cardiovascular), which must be treated to improve the quality of life of patients [209]. This approach focused on environmental optimization (education plans, treatment of comorbidities) despite not being curative and provided significant improvements [210]. It is evident that the results obtained depend on the severity of the disability, the type of treatments carried out and the age at which they are started. However, in many disorders such as epileptic encephalopathies, the development of cognitive deficits is difficult to prevent despite treatments.

## CONCLUSION

The treatment of epilepsy associated with ID remains a challenge for clinicians. Conventional ASMs still represent the mainstay of therapy, but new strategies are emerging. Hormone therapy has long been the first choice in the treatment of some epileptic disorders, such as West syndrome, and has shown encouraging results in some clinical trials. Non-pharmacological approaches play a leading role, especially in cases of drug resistance or drug toxicity. KD is a well-established treatment for many epilepsy syndromes such as Dravet syndrome, LGS, refractory infantile spasms and other drug-resistant epilepsies. Epilepsy surgery must be considered for seizures refractory to medical therapy and encompasses respective procedures and neuromodulation techniques. Finally, gene therapy has demonstrated a positive effect on seizure activity in preclinical studies and may play an important role in future epilepsy treatment. The main difficulties related to gene therapy consist in achieving stable, predictable and safe transgene expression. Deepening the knowledge of the molecular basis of epilepsy can lead to novel treatments directed to improving both the cognitive functioning and quality of life of patients.

## Figures and Tables

**Fig. (1):**
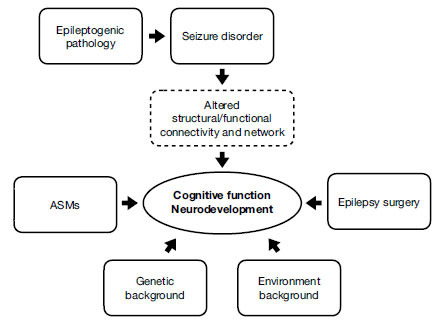
Multiple factors and their interaction that impact on cognitive function of children and adolescents with epilepsy.

**Table 1:** Principal gene alterations associated with epilepsy and intellectual disability.

**Authors**	**Genetic Alteration**	**Neurological Syndrome or Condition**	**Characteristics of Cognitive Impairment**
Kajal *et al*., 2011 [24]	TSC1, TSC2 MECP2, CDKL5 FMR1 ARX DCX NL1,NL3, NL4 NRP2	Tuberous Sclerosis Rett syndrome Fragile-X syndrome	Intellectual disabilities of varying degrees; language and motor delay; behavioral problems; symptoms of autism.
Chen *et al.*, 2017 [25]	Copy number variations: NRXN1, AUTS2, NLGN1, CNTNAP2, GRIN2A, PRRT2, NIPA2, BMP5 ion channels: LGT1, PRRT2, EFHC1, PRICKLE, RBFOX1, DEPDC5	-	Intellectual disabilities of varying degrees.
Stouffer *et al.*, 2016 [27]	Migration disorders: DCX, LYS1,ARX, TUBA1A, RELN, FLNA TUBB2B, GPR56, SRPX2 ASPM, MCPH1, STIL, GENPJ, WDR62, KIF5C	Lissencephaly Lissencephaly2/ Micropoligyry Microcephaly	Cognitive impairment, which ranges in severity from moderate to severe.
Verrotti *et al.*, 2018 [29]	1p36 monosomy	-	Developmental delay, intellectual disability rangin moderate-severe, hearing impairment, microcephaly.
Fu *et al.*, 2021 [30]	15q11-q13 microduplication	Angelman Prader-Willi	Developmental delay, moderate to severe intellectual disability, complete or partial absence of speech, problems with balance and coordination, excitability and psychomotor agitation, behavioral problem with aggression.
Khamirani *et al.*, 2021 [31]	STEGAL3	-	Intellectual disabilities of varying degrees.
Chen *et al.*, 2021 [32]	BRAF	Cardiofaciocutaneous Syndrome	Intellectual disability ranging from mild to severe.
Hofmeister *et al.*, 2021 [33]	SMARCA2	Nicolaides- Baraitser syndrome	Intellectual disability (in most cases severe, but also moderate or mild), language and motor delay; symptoms of autism.
Zanus *et al.*, 2020 [34]	SPATA5	-	Microcephaly, intellectual disability, sensorineural hearing loss, cortical visual impairment.
Coyan *et al*., 2020 [35]	3q29 microduplication	-	Mild or moderate intellectual disability and microcephaly.
Reynolds *et al.*, 2020 [36]	SCN2A	-	Intellectual disability, autism spectrum disorder.
Ponzi *et al.*, 2020 [37]	14q11-q22 microdeletion (FOXG1, NKX2-1, PAX9)	-	Intellectual disabilities of varying degrees.
Trivisano *et al*., 2020 [38]	SZT2	Epileptic encephalopathy	Macrocephaly, regression with increased seizures; mild to severe cognitive/intellectual delay; mild to severe delayed development.
Camp *et al.*, 2020 [39]	GRIN2D	-	All children have some degree of cognitive impairment, which ranges in severity from moderate to severe.
De Rinaldis *et al.*, 2020 [40]	EEFLA2	-	Intellectual disabilities of varying degrees.
Inuzuka *et al.*, 2020 [41]	SCN3A	Epileptic encephalopathy	Intellectual disabilities of varying degrees; the severity varies widely, ranging from isolated speech delay to severe developmental delay.
Trivisano *et al.*, 2019 [42]	PCDH19	-	Intellectual disabilities of varying degrees, behavioral disorders, autistic traits.
Shelkowitz *et al.*, 2019 [43]	IRF2BPL	-	Neurodegenerative disorders with regression and loss of motor skills and speech and abnormal movements.
Stamberg *et al.*, 2016 [44]	STXBP1	Epileptic encephalopathy	Intellectual disability severe-profound.
Romaniello *et al.*, 2019 [45]	TUBULIN family	-	Intellectual disability ranging from mild to severe.
Demarest *et al.*, 2019 [46]	CDKL5	Dravet Syndrome	Intellectual disability of various degrees, autistic traits, language difficulties, central visual impairment.
Ferretti *et al.*, 2019 [47]	POGZ	White-Sutton syndrome	Mild to severe intellectual disability, vision problems, features of autism spectrum disorder.
Fang *et al.*, 2019 [48]	20p13 microdeletion	-	Developmental delay, mild to moderate intellectual disability.
Bonnemason-Carrere *et al.*, 2019 [49]	PUM1	PADDAS syndrome	Developmental delay, moderate-severe intellectual disability, microcephaly.
Reijnders *et al.*, 2018 [50]	5q31.2 microdeletion	PURA syndrome	Moderate to profound intellectual disability, language impairment.
Agostini *et al.*, 2018 [51]	MED17	-	Progressive microcephaly, moderate intellectual disability, developmental delay, ataxic gait.

**Table 2:** Precision medicine in epilepsy.

**Mutated Gene**	**Encoded Protein**	**Epilepsy Syndrome(s)**	**Type of Mutation**	**Potential Therapy**
*GRIN2A*	NMDAR subunit	Various	Gain-of-function	NMDA receptor antagonists (memantine) [86]
*KCNT1*	Sodium-activated potassium channel	Nocturnal frontal lobe epilepsy EIMFS	Gain-of-function	Potassium channel openers (quinidine) [89]
*KCNQ2*	Voltage-gated potassium channel	Early-onset epileptic encephalopathy	Loss-of-function	Potassium channel openers (retigabine) [94]
*SCN1A*	Sodium channel Nav1.1	Dravet syndrome	Loss-of-function	Avoid sodium channel blockers [72]
*SCN2A*	Sodium channel Nav1.2	Epileptic encephalopathy EIMFS	Gain-of-function	Sodium channel blockers [95]
*SCN8A*	Sodium channel Nav1.6	Epileptic encephalopathy	Gain-of-function	Sodium channel blockers [96]
*TSC1, TSC2*	Hamartin, tuberin	Tuberous sclerosis complex	Loss-of-function	mTOR inhibitors (everolimus) [97]
*SLC2A1*	GLUT1	GLUT1-DS	Loss-of-function	Ketogenic diet [98]

## LIST OF ABBREVIATIONS

AAV: Adeno-associated virus
ADHD: Attention Deficit-hyperactivity Disorder
ADNFLE: Autosomal Dominant Nocturnal Frontal Lobe Epilepsy
AE: Edverse Effects
ASO: Antisense Oligonucleotides
BECTS: Benign Epilepsy with Centro-Temporal Spikes
CC: Corpus Callosum
CDM: Cortical Developmental Malformations
CFC: Cardio-facio-cutaneous
CSWS: Epilepsy with Continuous Spikes and Waves during Slow Sleep
CNV: Copy Number Variations
DBS: Deep Brain Stimulation
DEE: Developmentael and Epileptic Encephalopathies
DS: Dravet Syndrome
EIMFS: Epilepsy of Infancy with Migrating Focal Seizures
FDA: Food and Drug Administration
FXS: Fragile X Syndrome
GCE: Girls Clustering Epilepsy
GLUT1-DS: GLUT1 Deficiency Syndrome
GNX: Ganaxolone
ID: Intellectual Disability
IQ: Intelligence Quotient
IVIG: Intravenous Immunoglobulin
KD: Ketogenic Diet
LCTs: Long-chain Triglycerides
LGIT: Low Glycemic Index Treatment
LTG: Lamotrigine
MAD: Modified Atkins Diet
MCTD: Medium-chain Triglyceride Diet
MCTs: Medium-chain Triglycerides
NORSE: New-onset Refractory Status Epilepticus
RO: Regions of Overlap
SUDEP: Sudden Unexpected Death in Epilepsy
TCI: Transient Cognitive Impairment
TIQ: Total Intelligence Quotient
TLE: Temporal Lobe Epilepsy
TPM: Topiramate
TSC: Tuberous Sclerosis Complex
VNS: Vagus Nerve Stimulation
VPA: Valproic Acid
